# A pilot study to assess feasibility of value based pricing in Cyprus through pharmacoeconomic modelling and assessment of its operational framework: sorafenib for second line renal cell cancer

**DOI:** 10.1186/1478-7547-12-12

**Published:** 2014-05-03

**Authors:** Panagiotis Petrou, Michael A Talias

**Affiliations:** 1 HealthCare Management Program, Open University of Cyprus, 33 Giannou Kranidioti Avenue 2220, P.O BOX 12794, 2252 Nicosia, Cyprus

**Keywords:** JEL 110, JEL 130, JEL 300, Value based pricing, Pharmacoeconomic modelling, Sorafenib, Markov Model

## Abstract

**Background:**

The continuing increase of pharmaceutical expenditure calls for new approaches to pricing and reimbursement of pharmaceuticals. Value based pricing of pharmaceuticals is emerging as a useful tool and possess theoretical attributes to help health system cope with rising pharmaceutical expenditure.

**Aim:**

To assess the feasibility of introducing a value-based pricing scheme of pharmaceuticals in Cyprus and explore the integrative framework.

**Methods:**

A probabilistic Markov chain Monte Carlo model was created to simulate progression of advanced renal cell cancer for comparison of sorafenib to standard best supportive care. Literature review was performed and efficacy data were transferred from a published landmark trial, while official pricelists and clinical guidelines from Cyprus Ministry of Health were utilised for cost calculation. Based on proposed willingness to pay threshold the maximum price of sorafenib for the indication of second line renal cell cancer was assessed.

**Results:**

Sorafenib value based price was found to be significantly lower compared to its current reference price.

**Conclusion:**

Feasibility of Value Based Pricing is documented and pharmacoeconomic modelling can lead to robust results. Integration of value and affordability in the price are its main advantages which have to be weighed against lack of documentation for several theoretical parameters that influence outcome. Smaller countries such as Cyprus may experience adversities in establishing and sustaining essential structures for this scheme.

## Highlights

• Value based pricing is a new innovative way of pricing, which constitutes shifting from volume to value.

• We developed a probabilistic Markov Model to simulate disease progression of metastatic Renal Cell Cancer.

• We defined a price of sorafenib based on outcomes compared to best supportive care.

• Value based price of sorafenib is significantly lower compared to its current reference price.

• Many parameters have not been adequately addressed.

• It demands a multidisciplinary team which may be difficult for smaller countries to create and sustain.

## Introduction

Health policy makers worldwide have to cope with steadily increasing health care costs [1]. Pharmaceuticals constitute a significant part of this increase which has escalated into a major concern for the sustainability of health systems.

In the oncology sector, situation is even more crucial. In the USA, oncology medicines expenditure rose four fold in seven years [2] while in Cyprus, we observed a two fold increase of expenditure from 2005 to 2011 [3]. Most importantly, sales decomposition revealed that the dominant prescribing pattern in the oncology category is the shift from cheaper to more expensive new products.

Therefore new approaches to pricing and reimbursement of drugs are a prerequisite in order to maximise investment and reduce unjustified costs. At the same time, we must also safeguard the balance between static efficiency (acceptable cost/ benefit ratio) and the dynamic efficiency (promoting Research & Development -R&D) [4], since there are still unmet medical needs.

Currently, Cyprus applies external price referencing (EPR), the dominant pricing policy within European Union. For the scope of external price referencing, Drug Price Control Committee uses one expensive, two medium and one cheap EU countries. Simplicity of reference pricing makes it an ideal approach for smaller countries. These advantages come at the cost of lack of any theoretical basis and usually country selection is performed on secondary factors, such as geographical proximity and access to prices. In the WHO/HAI Project Medicine Prices and Availability [5] it’s stated that it’s doubtful whether the External Referencing Prices are “appropriate, efficient or optimal in accordance with any objective criterion”. Following countries blindfold trail behind reference ones and the risks of dissemination of flawed pricing approaches (too high or too low prices) is eminent.

Moreover EPR has some other drawbacks which may lead to price distortion:

• Many countries have implemented the clawback/payback schemes as a mechanism to avoid budget overshooting. Clawback / payback schemes [6] provide the recovery of amounts granted in a reimbursement system by payers given sales exceed a defined threshold. Therefore, companies still retain high price and avoid a price reduction which can escalate to a rolling spillover effect on the other reference countries. Clawback/ payback schemes alleviate the impact of high prices locally, but these schemes are not taken into consideration by the countries that reference.

• Selection of right price is trivial since products may carry many prices (retail, gross retail, reimbursement, ex-factory, official wholesale).

• The referencing system reaches a steady state following the convergence of prices and further reductions are not anticipated.

• Reference pricing does not reward innovation.

• It’s path dependant which results to heavily predictable outcomes.

In the context of aforementioned shortcomings of reference pricing, several health agencies have been experimenting with novel pricing approaches. Value based pricing(VBP) has emerged in Europe and two of the most important pharmaceutical market of Europe, UK and Germany, are shifting into Value based pricing.

### Value based pricing

Value based pricing constitutes a paradigm shift from volume to value. The aim is to convert the health benefits that the product delivers, which exceed the health benefits displaced in the broader health system and society due to additional cost incurred [7], into monetary value. The core of value based pricing is the incorporation of the product’s value into its price in the concept of a holistic pathway. It also safeguards access to effective and innovative drugs by setting a price that reflects the utility created [8]. From an industry perspective, this constitutes a clear motive to pursue innovation, which will be rewarded accordingly. From a payer’s perspective this leads to optimality of available resources.

Taking all the above into consideration, we designed a conceptual pilot study to assess practicability of adopting value based pricing in Cyprus from a payer’s perspective. We want to explore the feasibility of setting a price based on value. We track down all issues, positive and negative, stemming out of this process. Due to the fact that value based pricing is a new approach, several methodological and conceptual limitations exist. They include:

(a) The determination of affordability thresholds and overall affordability.

(b) The relative lack of identifying, measuring and valuing additional health benefits.

(c) Conversion from value to price.

(d) Data aggregation in heterogeneity population.

(e) Inherent challenges of measuring and comparing utilities of different types, different diseases and different stages of the same disease.

(f) Time lapse between availability of clinical data and best practice development.

(g) Ambiguity regarding optimal approaches of late external benefits that cannot be captured in the short term analysis [9].

Factors such as disease status and stage, bioethical arguments, inclusion or not of societal costs, uncertainty of results, robustness and reliability of clinical data infiltrate value definition and currently there is an ongoing debate regarding the actual definition [10].

## Methodology

We adopted the Consolidated Health Economic Evaluation Reporting Standards (CHEERS) (Table 1) guidelines for our economic evaluation [11].

**Table 1:** Cheers guidelines

**Item**	**Recommendation**	**Input**
1	Identify the study as an economic evaluation, or use more specific terms such as “cost-effectiveness analysis” and describe the interventions compared.	Value based pricing of Sorafenib compared to best supportive care.
2	Provide a structured summary of objectives, perspective, setting, methods (including study design and inputs), results (including base-case and uncertainty analyses), and conclusions.	Objectives, perspective, setting, methods (including study design and inputs), results including base-case and uncertainty analyses), and conclusions are included in the manuscript.
3	Provide an explicit statement of the broader context for the study. Present the study question and its relevance for health policy or practice decisions.	Definition of value based price for sorafenib. Definition of a price that reflects added value and utility of sorafenib treatment.
4	Describe characteristics of the base-case population and subgroups analyzed including why they were chosen.	Patients presented with metastatic RCC (as per indication)
5	State relevant aspects of the system (s) in which the decision (s) need (s) to be made.	Cyprus Public Health Care Sector
6	Describe the perspective of the study and relate this to the costs being evaluated.	Costs from Payer’s perspective in Cyprus
7	Describe the interventions or strategies being compared and state why they were chosen.	BSC vs sorafenib, a new VEGR agent product for this indication Bsc was chosed based on current local practice. Sorafenib was chosen since it belongs in the group of medicines with significant budget impact and high annual sales increase.
8	State the time horizon (s) over which costs and consequences are being evaluated and say why appropriate.	Time horizon is 10 years, by the end of this period all patients will transit into 3 stage (death).
9	Report the choice of discount rate (s) used for costs and outcomes and say why appropriate.	3.5% as per literature
10	Describe what outcomes were used as the measure (s) of benefit in the evaluation and their relevance for the type of analysis performed.	QALY due to its universal acceptance
11	Single study–based estimates: Describe fully the design features of the single effectiveness study and why the single study was a sufficient source of clinical effectiveness data.	A high quality low bias clinical trial [13]. In the Methodology section
12	Synthesis-based estimates: Describe fully the methods used for the identification of included studies and synthesis of clinical effectiveness data.	N/A
13	Model-based economic evaluation: Describe approaches and data sources used to estimate resource use associated with model health states. Describe primary or secondary research methods for valuing each resource item in terms of its unit cost. Describe any adjustments made to approximate to opportunity costs.	In the Methodology section
14	Report the dates of the estimated resource quantities and unit costs. Describe methods for adjusting estimated unit costs to the year of reported costs if necessary. Describe methods for converting costs into a common currency base and the exchange rate.	In the Methodology section
15	Describe and give reasons for the specific type of decision-analytical model used. Providing a figure to show model structure is strongly recommended.	In the Methodology section. Figure 1
16	Describe all structural or other assumptions underpinning the decision-analytical model.	In the Methodology section
17	Describe all analytical methods supporting the evaluation.	In the Methodology Section
18	Report the values, ranges, references, and, if used, probability distributions for all parameters. Report reasons or sources for distributions used to represent uncertainty where appropriate. Providing a table to show the input values is strongly recommended.	Tables 2, 3 and 4
19	For each intervention, report mean values for the main categories of estimated costs and outcomes of interest, as well as mean differences between the comparator groups. If applicable, report incremental cost-effectiveness ratios.	In Results section. Table 5
20	Model-based economic evaluation: Describe the effects on the results of uncertainty for all input parameters, and uncertainty related to the structure of the model and assumptions.	Sensitivity analysis was performed. Table 6
21	Summarise key study findings and describe how they support the conclusions reached. Discuss limitations and the generalisability of the findings and how the findings fit with current knowledge	In Results section

Sorafenib, a Vascular Endothelial Growth Factor Inhibitor (VEGF), was selected for the indication of second line renal cell cancer (RCC) [12], since this therapeutic category demonstrates significant sales increase which exerts considerable pressures on pharmaceutical expenditure [3]. Due to the current financial recession and the bailout agreement between Cyprus and Troika, there is a need to assess all cost drivers, especially agents associated with significant cost and clinical uncertainty. Moreover, innovative status of sorafenib implies that cost-containment approaches such as tendering and internal price referencing are relatively ineffective. As a comparative arm, we have chosen best supportive care (BSC), according to local guidelines in Cyprus. Although European Society of Medical Oncology (ESMO) recommends axitinib and pazopanib for second line treatment of renal cell carcinoma [13], current recession and austerity measures impede introduction of new products in the formulary.

**Figure 1:**
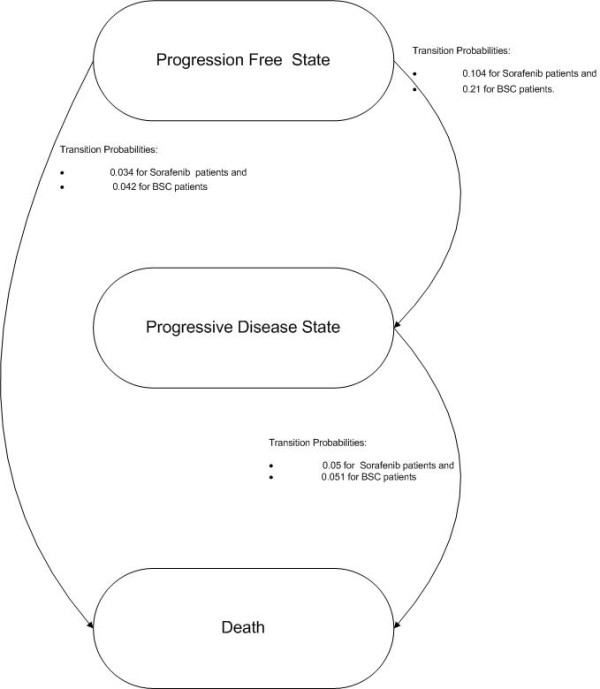
Markov model for second line m RCC.

We define a probabilistic Markov analytical decision Model which simulates disease progression [14,15] in RCC. The Markov Model (Figure 1) is a memoryless process which describes the evolution of disease between health states in a stochastic way based on the transition probabilities [16], which depend only on the current state of the process and not on previous states. Three non- absorbing health states were identified: Progression-free survival (PFS), Progression disease (PD) and death. Patients are supposed to enter the model in PFS state, after their diagnosis with metastatic RCC is confirmed (Figure 1). Due to low life expectancy of these patients, we assume that each cycle lasts one month and therefore the transition probabilities are also defined per month [17]. Model was synthesized in Winbugs 1.4.3 (Windows Bayesian Inference Using Gibbs Sampling) [18], a software for specifying complex Bayesian models [19]. Benefits of Bayesian methodology have been extensively documented by many authors [20-24].

**Figure 2:**
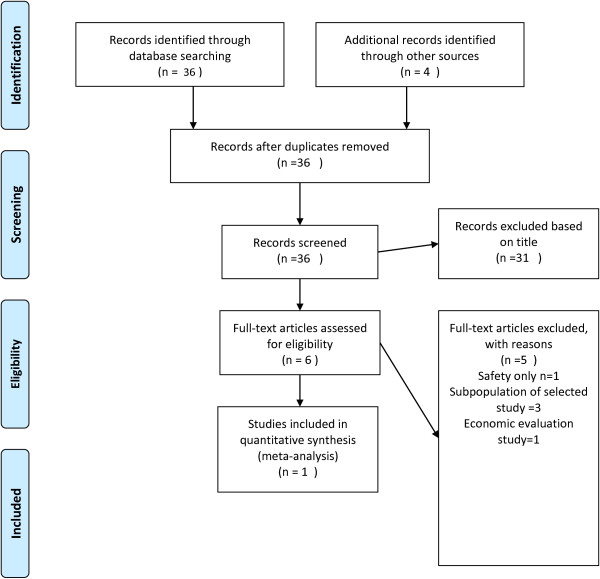
Flow Diagram of literature review of Sorafenib in Second line renal cell carcinoma.

We performed a literature review using mesh terms “Sorafenib” “Carcinoma, Renal Cell” and “Randomised Controlled trial” (Figure 2).

Literature review tracked down 36 studies eligible for inclusion. We identified only one study that compares sorafenib with BSC, which was also the unique study for BSC [25]. TARGET trial is a large phase 3, high quality and low bias study trial (Figure 2). This is a multicenter, multinational, randomised double blind clinical trial and it was also used for assessment of sorafenib by NICE [12]. This study demonstrated the survival benefit of sorafenib over BSC [26], lasted for one and a half years and recruited 903 patients with renal cell carcinoma that was resistant to standard therapy. Eighty three percent of recruited patients received cytocine therapy as first line. The median age of patients in this trial was 58 years. Sorafenib was significantly superior compared to BSC for both PFS and overall survival (OS): For PFS, the hazard ratio (HR) was 0.51 (95% confidence interval [CI]: 0.43–0.60), and for OS, the HR was 0.72 (95% CI: 0.54–0.94). Based on the Progression free and Progression disease duration, we estimated the transition probabilities which will be incorporated in the Markov model, according to the following approach:

Riskofanevent1month=1-0.5^1/mediantimetoevent

[27]

This can be easily derived through the equations:

$$\begin{array}{l} P=1-e^{–R}\mathrm{and}\\ R=-\ln\;\left[0.5\right]/\left(\mathrm{Time}\;\mathrm{to}\;\mathrm{event}/\mathrm{number}\;\mathrm{of}\;\mathrm{treatment}\;\mathrm{cycles}\right) \end{array}$$

[28]

Monthly transition probabilities to progressive disease were defined as:

• 0.104 for Sorafenib patients and,

• 0.21 for BSC patients.

Monthly death probabilities (from progression free state) were defined as:

• 0.034 for Sorafenib patients and,

• 0.042 for BSC patients.

Monthly death probabilities (from progressive state) were defined as:

• 0.05 for Sorafenib patients and,

• 0.051 for BSC patients.

In order to incorporate uncertainty in the model, we expressed these probabilities as beta distributions [29,30]. Beta distribution is defined as beta (*α*, β) and *α* denotes number of patients that transit to next stage while *β* is the total sample size minus number of patients who shift to the next disease stage [31,32]. We set the time horizon as a decade by the end of which all patients will shift into 3^rd^ stage. Due to absence of any official guidance regarding technical parameters, we set discounting rate at 3.5% according to current practice in UK [33] and Sweden [34]. Willingness to pay (WTP) threshold is not officially defined in Cyprus. Therefore, we adopted recommendations of WHO [35] regarding utilisation of multiplies of Gross Domestic Product (GDP) per capita as a proxy for WTP threshold. In 2012 the estimated GDP per capita current prices was 20,517 euro [36], therefore we define the WTP threshold at 60,000 euro. Since sorafenib is an orphan drug, we used the highest recommended level. We performed one way sensitivity analysis in order to identify which variables and to which extent affect outcome.

Cost distribution of general medical and other pharmaceutical costs (Table 2) (excluding sorafenib cost) was assumed to follow gamma distribution since typically costs are non- normally distributed, highly skewed and demonstrate kurtosis [37]. Method of moments [38] was applied in order to estimate parameters of this distribution.

**Table 2:** Distribution for cost

	**BSC ARM**	**Medical and other pharmaceutical cost in progression free stage**	**Progression stage**	**Sorafenib arm**	**Sorafenib cost**	**Medical and other pharmaceutical cost in progression free stage**	**Cost in the progression stage 1**^**st **^**and 2**^**nd **^**month**	**3**^**nd **^**Month and further on**
Cost (euro) 2012		278	770		2880	357	1499 ^1^	770
Type of distribution		Gamma	Gamma		Uniform	Gamma	Gamma	Gamma
Distribution parameters α, β		(1336, 4.8)	(3696, 4.8)		(2880,2900)	(1714, 4.8)	(7196, 4.8)	(3696, 4.8)

^1^Provides that patients will continue sorafenib for one month after progression until diagnosis is confirmed.

Sorafenib costs were denoted by a uniform distribution as per the recommended (approved) daily dosage since we assume that all patients receive recommended daily dose. We adopted the health state utilities as reported by Thomson [12] which were assessed through the use of UK EQ-5D: health state utilities of 0.76 (s.e. 0.03) for PFS and 0.68 (s.e. 0.04) for PD. Since utility value are defined between 0 and 1, we assume that they follow a beta distribution as following [39,40]:

• Progression Free State (153.26, 48.4),

• Progressive disease state (91.8,43.2).

Markov Model was loaded with an initial cohort of 1,000 patients. Patients were supposed to be on the second line of treatment with sorafenib with an indication of metastasis. We discarded the first 50,000 iterations of simulation to ensure stability of the model and we performed another 50,000 iterations to ensure convergence and accuracy of data. We checked convergence through trace plots of samples and standard error of the results.

### Other medical and pharmaceutical costs

Cancer patients in Cyprus, with annual income less than 150,000 euro are entitled to free public medical care. Utilization of pharmaceutical and medical cost was assessed based on National guidelines and availability of products listed in the national formulary. Costs of pharmaceuticals (sorafenib and adjuvant pharmaceutical care) were extracted from the national formulary while medical costs were calculated based on 2012 database [41,42] (Tables 3 and 4).

**Table 3:** Health services use and costs

**Parameter**	**PFS**		**PD**
	**Consultation**	**CT scan**	**Consultation**
Sorafenib	1 specialist visit €40	€256 (every 3 months)	1 GP 2 nurses 1 psychologist €70
	Annual costs related to hypertension: 3 visits € 60		
BSC	1 GP 2 nurses 1 psychologist €70	€256 (every 6 months)	1 GP 2 nurses 1 psychologist €70
Hospitalization (Daily)	€135		€135
Blood test s (Full blood count, liver function SGPT SGOT and creatinine	€157		€157

Monthly Costs (unless otherwise specified).

**Table 4:** Pharmaceutical costs

	**Antihypertensive Therapy**	**ACE inhibitors**	**Amlodipine 5 mg**	**Losartan 50 mg**	**Opiods**	**Ondansetron 8 mg**	**Fentanyl 50 mcg**	**Fentanyl 25 mcg**	**Fentanyl 100 mcg/hr**	**Morphine 30 mg**	**Morphine 20 mg**	**Morphine 10 mg 1 tab**	**Morphine 10 mg/ml**
Dosage		o.d	o.d	o.d		Per Need	1 patch every 72 hours	1 patch every 72 hours	1 patch every 72 hours	30 b.i.d	20 b.i.d.	Up to 100 bid	5-20 mg per 4 hours
Cost per unit (tab, vial, patch)		0.02	0.02	0,06		0,66	10,43	5,4	19	0,19	0,33	0,07	0,36
Monthly cost		0,6	0,6	1,8			10,43	54	190	11,4-34.2	19,8 -99	4,2 -54	54

## Results

Treatment with sorafenib results to incremental gain 0,1605 QALY per patient, compared to BSC. This would lead to 16996 euro cost per sorafenib patient (CI 95%: 13140-18950) compared to 7336 euro per patient on best supportive care (CI 95%: 6327.0- 8468.0). Our Value based pricing approach indicates that under a 60,000 theoretical WTP threshold, price of sorafenib should be set at 1816 euro per package, a price notably lower compared to current price. Under current price (2880 per package) the Incremental Cost Effectiveness Ratio (ICER) is 102,879 and the health gains cost 16470 euro additional per patient (Table 5).

**Table 5:** Threshold leves and Corresponding value based price of Sorafenib

**Willingness to pay Threshold**	**20,000**	**40,000**	**60,000**	**>100,00 (102,616)**
**Cost of sorafenib arm**	10620 (CI 95% 9022.0-12490.0)	13760 (CI 95% 11680.0-16290.0)	16996 (CI 95%14370.0-20120.0)	23806 (CI 95% 20,000 -28220)
**Cost of bsc arm**	7336.0 (CI 95%: 6327.0- 8468.0).	7336.0 (CI 95%: 6327.0- 8468.0).	7336.0 (CI 95%: 6327.0- 8468.0).	7336.0 (CI 95%: 6327.0- 8468.0).
**Incremental QALY gains**	0,1605 QALY	0,1605 QALY	0,1605 QALY	0,1605 QALY
**Incremental cost**	3284	6424	9630	16470
**VBP of sorafenib**	810	1325	1816	2880

### Sensitivity analysis

We performed one way sensitivity analysis. ICER was proved to be significant sensitive to the price of sorafenib, while medical and other pharmaceutical had a minimum impact on ICER. ICER is also sensitive to utilities and to PFS while it’s less sensitive to OS (Table 6).

**Table 6:** Sensitivity analysis

**Parameter**	**Baseline value**	**Sensitivity analysis**	**New price**	**ICER**	**Base case**
Sorafenib price	1816 (per month)	50% reduction	908	24,190	60,000
TIME HORIZON	10 YEARS	5 YEARS	1860	60,266	60,000
Discounting	3.5	0	2455	45,279	60,000
Discounting	3.5	1.5	2124	51,025	60,000
Discounting	3.5	5	1695	67,203	60,000
QALY	0.76 – 0.68	0,836 0.748	2013	54,738	60,000
QALY	0.76 – 0.68	0.684- 0.612	1711	66,863	60,000
Medical and other pharmaceutical costs		Increase 20%	1926	57,407	60,000
Medical and other pharmaceutical costs		Decrease 20%	1802	62,282	60,000
Increase of PFS and OS 10%			2030	53,300	60,000
Increase of OS 10%			1905	58,329	60,000
Increase of PFS 10%			1987	55,701	60,000
Decrease of PFS and OS 10%			1580	72,374	60,000
Decrease of OS 10%			1790	62,695	60,000
Decrease of PFS 10%			1655	68,853	60,000

## Discussion

Health care costs are rapidly expanding. Several cost containment approaches such as price reductions, internal price referencing, tendering and risk-sharing have been applied extensively. Although undisputedly potent in the short-term, tendering and price reduction approach lack selectivity and both oversee product’s innovation level and particularities of involved patient categories. On the other hand, risk sharing schemes were implemented to tackle uncertainty primarily for short term and due to their binary evaluation context they tend to benefit insurer and sustainability is severely doubted [43]. Therefore they cannot be considered as a long term approach. Other current pricing schemes such as EPR do not promote innovation, while some authors argue that EPR leads to high prices of medicines which are not aligned to their value [44].

Value based pricing is a paradigm shift that distributes risk among payer and industry and offers measurable value to payers. Our approach indicates that value based pricing is a feasible approach in Cyprus Health sector. Nevertheless, several practical issues were raised during the procedure that have to be tackled before value based pricing is disseminated. We identified only one clinical study, whose design matches current practice in Cyprus, nevertheless this would not be the norm for the majority of drugs. Marketing authorization holders run randomised controlled trials for regulatory purposes, whose design deviates from real life settings due to comparator choice, exclusion and inclusion criteria, patient population, duration of the trials, setting, outcome measures and duration. Assessing data and synthesizing relevant models for economic evaluation raise substantially complexity factor and this constitutes value based pricing a lengthy, labor and expertise demanding process. It would demand strong support and commitment by government and mainly a multidisciplinary pool of people with the appropriate health economic, statistical and epidemiological skills. A small country such as Cyprus may struggle to maintain the necessary human resources required especially given current financial recession. Another obstacle emerging from size of Cyprus deals with maximum output capacity. A proper economic evaluation may span up to one year, and it’s doubtful whether current Health context can support more than one committee. Therefore, relevant output capacity of this committee will be low and full market coverage is illusive. Consequently it’s expected that given that such a committee is assembled, it can focus only on selected products, with significant disease or budget impact. This could create inequalities among pharmaceutical market and create 2 tier products.

Cyprus, due to its small size and remote location, is classified as a non-attractive small pharmaceutical market [45]. A distinct characteristic of this market is the existence of low competitive forces, which tend to shift monopoly power to supplier. Supplier’s monopoly power is also augmented by entry barriers, such as obligations of marketing authorization holder to supply summary of product’s characteristics in Greek, along with Greek labeled packages. Being a small market deters the development of alternative supply chains, such as parallel imports, which could have compromised dominant position of a single supplier. Therefore, we would need to assess potential exit of pharmaceutical industries from Cyprus, in case of price reductions. However, this has to be weighed against a much faster introduction of products to the formulary.

Sorafenib has two indications, renal and liver cancer. Under proposed value based pricing, this will create severe implications since potentially sorafenib will carry 2 prices, which will further increase complexity factor of reimbursement process. This may lead to a weighted value based pricing, based on estimated utilization data.

Establishment of a WTP threshold remains uncharted territory for many countries primarily due to ethical reasons and the decision making is performed on the basis of unpublished pertinent thresholds. Cost effectiveness has a comparative and a limiting context since all medicines would be cost effective under an infinitely large WTP threshold. For our study we adopted recommendations of WHO, which provides that multiplies of per capita domestic product can be used as economic threshold. More importantly, this approach takes into consideration financial capacity of each country. According to this approach, three time per capita domestic product is the highest WTP threshold; anything above this is considered to be non- cost effective and resources could create more health utility diverted to other therapeutic territories. This is in line with differential price concept and in contrast to external price reference, this allows affordability of each country to contribute to prices of pharmaceuticals.

Some authors take a step forward and suggest the introduction of varying thresholds level: each one addressing a specific health state under the condition that ethical and legal concerns can be addressed. A higher WTP threshold would probably suit better condition with greater burden of illness, such as rare and orphan diseases, end of life treatment, highly innovative products and medicines that exhibit wider societal benefits, such as benefits to carers [46]. Many authors argued about potential extra weight of QALY in end of life treatments [47] while others debate that even a QALY at the end of life actually varies according [48] to the way it was obtained, with gain in palliative care being superior [49,50] to gains in life expectancy. Since all health programs actually compete for funds it’s possible that this diversity may be beneficial for some patients and injurious for others. Ginette Camps-Walsh [7] suggests 5 different categories of threshold within NHS which differentiate acute, chronic, paediatric, rare and end of life diseases. The categories above have varying degrees of treatment options and as a result, each category has diverse unmet medical needs.

Capitalising on this, we adopted the higher WTP threshold, due to orphan drug status of sorafenib.

The above issue is also linked to utilisation of different health state measure tools. It’s accepted that available health state measurement tools [51] can deliver varying results and it’s also documented that patients in different stages of the same disease have different perception of time [52] and health state preferences [53].

These findings create further complications regarding the selection of endpoints of the study (Overall survival or Progression free survival) which must be consistent in order to ensure homogeneity among potentially comparative products.

Comparator selection and specifically the base care product, is of paramount importance. In a time series setting, the price of future products will be a step-up dependant based on past and current value based prices. In our case, we compared sorafenib to BSC, with BSC being the base case product. Upon future introduction of axitinib, its price will greatly depend on price of sorafenib and there will be notable differences between sorafenib’ s reference (2880 euro) and sorafenib’ s value based price (1816 euro).The level of complexity will further rise given that in oncology regimens, it’s not rare to encounter expensive products, apart from primary ones, which are given as adjuvant or to cure side effects. It’s still unknown how to address this issue regarding products that were priced ex post and products that will be priced ex ante.

Another decisive task is to express all values into money: Some authors suggest the net-benefit while other authors argue for the use of multicriteria decision analysis, by using weight value for each benefit type [54].

Value based pricing is expected to engage R & D companies in a quest for really innovative products, but it may deter companies from investing into territories, in which marginal benefits are anticipated [55]. Another pending issue is the pricing of equivalent products and the concern that this will impede further price competitions which have led to massive reductions in some therapeutic categories, such as statins [56].

As proved by our analysis value based pricing does not result in high pharmaceutical prices when society’s WTP is known and under a specific context it can be considered a cost containment tool [57]. This does not come under surprise since oncology products due to their innovative mode of action status, high R & D costs and considerable failure rates ask for higher prices.

In our study we transferred health utilities from published study. Value based pricing framework in other countries, such as Germany provides that a product gets a provisional price, and afterwards “real life effectiveness data” [58] are gathered, which will be utilized to set a value based price [59]. For new products this preferably has to be carried out in national level. This is in line with other approaches which provide that new products get a price based on an *ex ante* evaluation while existing products get a price based on a rolling *ex post* evaluation [60].

## Conclusions

A value based pricing scheme is feasible in Cyprus. Estimated value based price of sorafenib is significantly lower, which is in line with findings of other authors [61].

Although many issues are still pending, incorporation of value and affordability into the product price, comprise essential rationale for its further dissemination. Industry and health authorities must engage in a dialogue to crystallize all aspects since despite its potentials, value based pricing displays a high complexity factor.

## Abbreviations

VBP: Value based pricing; MoH: Ministry of health; R & D: Research and development; PFS: Progression free survival; PD: Progressive disease; OS: Overall survival; RCC: Renal cell cancer; BSC: Best supportive care.

## Competing interests

The authors declare that they have no competing interests.

## Authors’ contributions

PP participated in the design and coordination of the study and drafted the manuscript. This manuscript is part of a PhD thesis. MT helped to draft the manuscript and is a supervisor of the thesis. Both authors read and approved the final manuscript.
